# Edge co-occurrences can account for rapid categorization of natural versus animal images

**DOI:** 10.1038/srep11400

**Published:** 2015-06-22

**Authors:** Laurent U. Perrinet, James A. Bednar

**Affiliations:** 1 Institut de Neurosciences de la Timone, CNRS / Aix-Marseille Université; 2 Institute for Adaptive and Neural Computation, University of Edinburgh.

## Abstract

Making a judgment about the semantic category of a visual scene, such as whether it contains an animal, is typically assumed to involve high-level associative brain areas. Previous explanations require progressively analyzing the scene hierarchically at increasing levels of abstraction, from edge extraction to mid-level object recognition and then object categorization. Here we show that the statistics of edge co-occurrences alone are sufficient to perform a rough yet robust (translation, scale, and rotation invariant) scene categorization. We first extracted the edges from images using a scale-space analysis coupled with a sparse coding algorithm. We then computed the “association field” for different categories (natural, man-made, or containing an animal) by computing the statistics of edge co-occurrences. These differed strongly, with animal images having more curved configurations. We show that this geometry alone is sufficient for categorization, and that the pattern of errors made by humans is consistent with this procedure. Because these statistics could be measured as early as the primary visual cortex, the results challenge widely held assumptions about the flow of computations in the visual system. The results also suggest new algorithms for image classification and signal processing that exploit correlations between low-level structure and the underlying semantic category.

Oriented edges in images of natural scenes tend to be aligned in co-linear or co-circular arrangements, with lines and smooth curves more common than other possible arrangements of edges. The visual system appears to take advantage of this prior knowledge about natural images, with human contour detection and grouping performance well predicted by such an “association field”^1^ between edge elements. Geisler *et al.*^2^ have estimated this prior information available to the visual system by extracting contours from a database of natural images, and showed that these statistics could predict behavioral data from humans in a line completion task.

One possible candidate substrate for implementing an association field in mammals is the set of long-range lateral connections between neurons in the primary visual cortex (V1), which could act to detect contours matching the association field^3^. To fill this role, lateral connections would need to be orientation specific and aligned along contours^4^, and indeed such an arrangement has been found in V1 of the tree shrew^3^,^5^ and the monkey^6^.

In this paper, we show that an association field of this type can be used for image categorization. Human performance in tasks like determining whether an image contains an animal is surprisingly accurate even at very rapid time^7^. To explain this rapid process, previous researchers have investigated whether low-level features could explain human performance in rapid categorization tasks, but they concluded with general claims that “it is very unlikely that human observers could rely on low-level cues”, (SI Table 2)^8^, and “low-level information [alone] cannot explain human performance”, (p.19)^9^. Here we show that alternative low-level cues, namely the association field between edges represented as early as V1, can achieve image categorization performance comparable to humans and to previous hierarchical models of the visual system. We also show the images falsely reported as having animals by humans have association fields strongly resembling those of animal images, suggesting that humans are making use of this co-occurrence information when performing rapid image categorizations.

## Results

**Figure 1:**
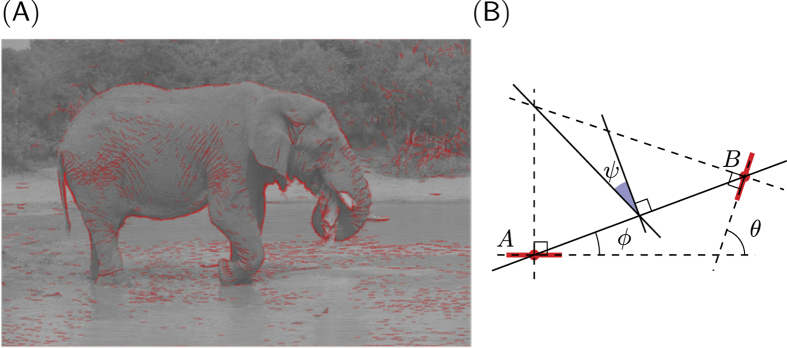
(**A**) An example image with the list of extracted edges overlaid. Each edge is represented by a red line segment which represents its position (center of segment), orientation, and scale (length of segment). We controlled the quality of the reconstruction from the edge information such that the residual energy was less than 5%. (**B**) The relationship between a reference edge *A* and another edge *B* can be quantified in terms of the difference between their orientations *θ*, ratio of scale *σ*, distance *d* between their centers, and difference of azimuth (angular location) *ϕ*. Additionally, we define *ψ* = *ϕ* − *θ*/2, which is symmetric with respect to the choice of the reference edge; in particular, *ψ* = 0 for co-circular edges. As in Geisler *et al.*^2^, edges outside a central circular mask are discarded in the computation of the statistics to avoid artifacts. Image credit: Andrew Shiva, Creative Commons Attribution-Share Alike 3.0 Unported license.

To determine what information edge co-occurences could provide about image category, we examined how the statistics of edge co-occurrence vary across three image categories. The first two consist of the image databases (600 images each) used by Serre *et al.*^8^, which contain either animals at different close-up views in a natural setting (which we call “animal images”), or natural images without animals (which we call “non-animal natural images”). A third database consists of self-acquired images from a biology laboratory setting, containing 600 indoor views of a man-made indoor environment in which animals are reared (which we call “man-made images”). These images also do not contain animals, but provide a novel set for control purposes. From a sparse representation of oriented edges at different scales, we define the association field as the four-dimensional histogram of edge co-occurrences *p*(*d, ψ, θ, σ*) (see definitions in Fig. 1).

**Figure 2 f2:**
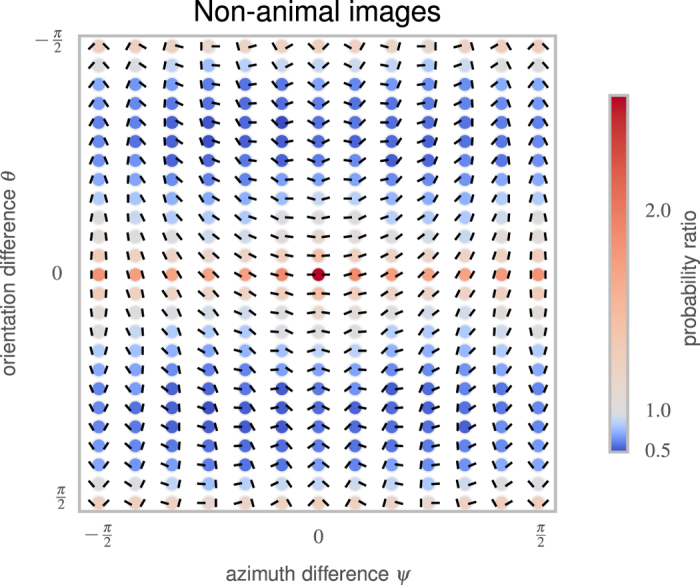
The probability distribution function *p*(*ψ,θ*) represents the distribution of the different geometrical arrangements of edges’ angles, which we call a “chevron map”. We show here the histogram for non-animal natural images, illustrating the preference for co-linear edge configurations. For each chevron configuration, deeper and deeper red circles indicate configurations that are more and more likely with respect to a uniform prior, with an average maximum of about 3 times more likely, and deeper and deeper blue circles indicate configurations less likely than a flat prior (with a minimum of about 0.8 times as likely). Conveniently, this “chevron map” shows in one graph that non-animal natural images have on average a preference for co-linear and parallel edges, (the horizontal middle axis) and orthogonal angles (the top and bottom rows), along with a slight preference for co-circular configurations (for *ψ* = 0 and *ψ* = ± 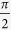, just above and below the central row).

**Figure 3:**
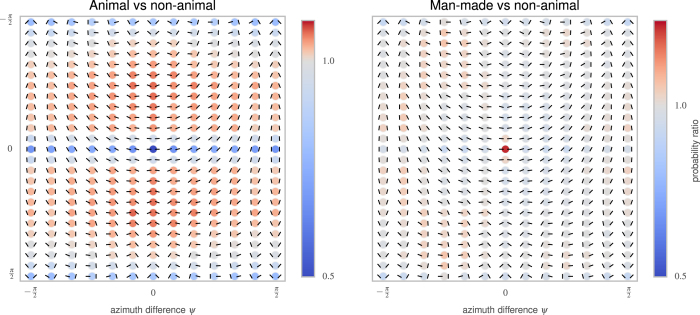
As for Fig. 2, we show the probability of edge configurations as chevron maps for two databases (man-made, animal). Here, we show the ratio of histogram counts relative to that of the non-animal natural image dataset. Deeper and deeper red circles indicate configurations that are more and more likely (and blue respectively less likely) with respect to the histogram computed for non-animal images. In the left plot, the animal images exhibit relatively more circular continuations and converging angles (red chevrons in the central vertical axis) relative to non-animal natural images, at the expense of co-linear, parallel, and orthogonal configurations (blue circles along the middle horizontal axis). The man-made images have strikingly more co-linear features (central circle), which reflects the prevalence of long, straight lines in the cage images in that dataset.

Computing the Kullback-Leibler (KL) divergence between this four-dimensional function and various possible factorizations suggests that we can consider *p*(*d, σ*) separately from *p*(*ψ, θ*) (see SI Table 1). The distribution of edge distances and relative scales *p*(*d, σ*) proved to be quite similar across the different classes of images[Fig f2] (see Fig. 3), because these variables depend primarily on the viewpoint and location of the observer rather than on the objects in the scene. The remaining two angular parameters *p*(*ψ, θ*) can be visualized using a “chevron map” (see Fig. 2), which indicates the probability of each possible angular configuration between edges. As found in previous work^2^, collinear, parallel, and (to some extent) orthogonal configurations appear more commonly than chance. Results for other datasets are broadly similar, but with systematic differences. Figure 3 shows chevron maps for the difference between the non-animal natural image dataset and the other two datasets. In particular, images of man-made environments have more collinear configurations, while images with animals have more highly curved and converging angles, and fewer collinear or orthogonal configurations.

**Figure 4:**
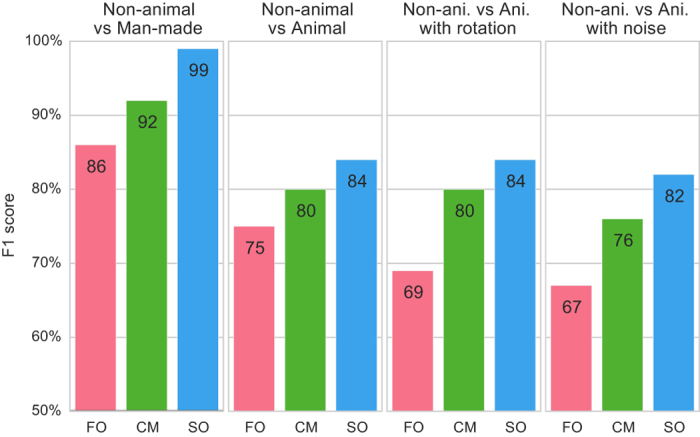
Classification results. To quantify the difference in low-level feature statistics across categories (see Fig. 3), we used a standard Support Vector Machine (SVM) classifier to measure how each representation affected the classifier’s reliability for identifying the image category. For each individual image, we constructed a vector of features as either (FO) the histogram of first-order statistics as the histogram of edges’ orientations, (CM) the “chevron map” subset of the second-order statistics, (i.e., the two-dimensional histogram of relative orientation and azimuth; see Fig. 2), or (SO) the full, four-dimensional histogram of second-order statistics (i.e., all parameters of the edge co-occurrences). We gathered these vectors for each different class of images and report here the results of the SVM classifier using an F1 score (50% represents chance level). While it was expected that differences would be clear between non-animal natural images versus laboratory (man-made) images, results are still quite high for classifying animal images versus non-animal natural images, and are in the range reported by Serre *et al.*^8^ (F1 score of 80% for human observers and 82% for their model), even using the CM features alone.

To assess these differences quantitatively, we built a simple classifier to measure if this representation is sufficient to categorize different image categories reliably. For each individual image, we constructed a vector of features as either (FO) the first-order statistics, i.e., the histogram of edge orientations, (CM) the “chevron map” subset of the second-order statistics, (i.e., the two-dimensional histogram of relative orientation and azimuth; as in Fig. 2), or (SO) the full, four-dimensional histogram of second-order statistics (i.e., all four parameters of the edge co-occurrences). We gathered these vectors for each different class of images and tested a standard Support Vector Machine (SVM) classification algorithm. The results of the SVM classifier are reported using an F1 score, as in Serre *et al.*^8^, which equally weights false positive and false negative error rates to fairly assess each approach. Here we used the F1 score to directly compare our method to that of Serre *et al.*^8^. This process was cross-validated 20 times by drawing new training and testing sets. Using these different trials, we could measure the variability of the F1 score. The variability was always less than ≈ 4%. Results are summarized in Fig. 4.

Performance is almost perfect for distinguishing non-animal natural versus laboratory (man-made) images, and is still quite high for classifying animal images versus non-animal natural images, a much more subtle distinction. This high level of performance is very surprising, given the explicit claims from Serre *et al.*^8^ and others that no low-level cue was likely to work well. Results for the chevron map confirm that performance of the classifier comes primarily from a geometrical feature rather than a viewpoint-dependent feature. Note that by definition, our measure of the statistics of edge co-occurrence is invariant to translations, scalings, and rotations in the plane of the image. This last property—not shared by first-order statistics of edges—makes it possible to explain the rather unintuitive result that categorization in humans is relatively independent to rotations^10^. We also performed the same classification where images’ signal-to-noise ratio was halved (“Noise” condition above). Results are degraded but qualitatively similar, as was also observed in psychophysical experiments^11^.

**Figure 5:**
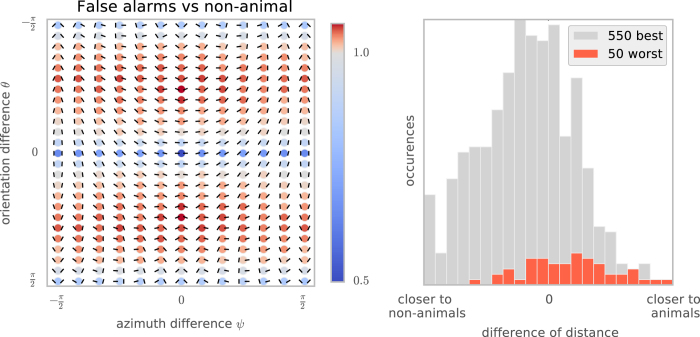
To see whether the patterns of errors made by humans are consistent with our model, we studied the second-order statistics of the 50 non-animal images that human subjects in Serre *et al.*^17^ most commonly falsely reported as having an animal. We call this set of images the “false-alarm image dataset”. (Left) This chevron map plot shows the ratio between the second-order statistics of the false-alarm images and the full non-animal natural image dataset, computed as in Fig. 3 (left). Just as for the images that actually do contain animals (Fig. 3, left), the images falsely reported as having animals have more co-circular and converging (red chevrons) and fewer collinear and orthogonal configurations (blue chevrons). (Right) To quantify this similarity, we computed the Kullback-Leibler distance between the histogram of each of these images from the false-alarm image dataset, and the average histogram of each class. The difference between these two distances gives a quantitative measure of how close each image is to the average histograms for each class. Consistent with the idea that humans are using edge co-occurences to do rapid image categorization, the 50 non-animal images that were worst classified are biased toward the animal histogram (*d*′ = 1.04), while the 550 best classified non-animal images are closer to the non-animal histogram.

Finally, if humans use edge co-occurences to do rapid image categorization, images falsely detected by humans as containing animals should have second-order statistics similar to those of images that do contain animals. Figure 5 shows that the set of the most common false-alarm images does have a chevron map strikingly similar to that for images that do have animals, and that on average the false alarm images have second-order histograms closer to the animal than to the non-animal datasets.

## Discussion

These results call into question previous claims that a hierarchical analysis of the visual scene is necessary for classification into high-level categories^8^. We speculate that the observed differences in second-order statistics for images with animals have an underlying basis in the physical constraints governing the shapes of animals. Specifically, animals typically have compact shapes, constrained by their capacity to move, unlike plants rooted in one location that must stretch rather than move towards resources. Conversely, man-made objects tend to have long, straight lines due to their methods of manufacture. We would expect that other categories of objects could similarly be distinguished by their second-order statistics, assuming that their form follows their function in ways analogous to the categories tested here. Thus we expect that the second-order statistics will be useful as a rough but general and fast method for distinguishing a wide range of scene and object categories. Similar observations apply to other sensory systems, where we would expect co-occurence between primary sensory elements (such as stimulation of a patch of skin, presence of a specific auditory frequency, or activation of a taste or smell receptor) to differ between ecologically important classes of stimuli.

In this study, we showed that edge co-occurrences were sufficient to distinguish between the animal/non-animal datasets from Serre *et al.*^8^ and Kirchner and Thorpe^12^, with performance comparable to that of humans in rapid categorization tasks. We also showed that these statistics can distinguish between these datasets and scenes of various man-made environments. How well will this approach generalize to other datasets, such as different combinations of animal/non-animal datasets? Our analysis suggests that similar performance should be found for all dataset pairs that have a statistically significant difference in the “roundness” of the contours extracted in the images. Although we expect such differences to be found reliably across the animal/non-animal datasets currently in use, it should be possible to find or construct a non-animal dataset that has similar edge co-occurence statistics to that of an animal dataset. For such comparisons, the model predicts that human observers would also have trouble rapidly making such a distinction (as suggested by the similar patterns of errors in Fig. 5). Selecting or constructing such image sets and testing them with human observers will be an important way that the performance of this approach can be tested in future studies; even though humans should be able to categorize the images reliably when given enough time for study, the model predicts that they will be unable to do so under the constraints of rapid categorization.

In addition, our results predict that animal measurements of *p*(*θ, ψ*) should be dynamically adaptive, as recently reported by McManus *et al.*^13^ for macaque V1, since *p*(*θ, ψ*) varies strongly across environments. The statistics of the dense network of lateral connections in V1 analyzed by Bosking *et al.*^5^ and Hunt *et al.*^3^ suggest that a local representation of these probabilities is available for supporting such computations. We predict that if these patterns of connectivity are built by adapting to the visual statistics, e.g., through Hebbian learning^14^, lab-reared animals will have much stronger connections between neurons with collinear preferences than will wild-raised animals. Finally, a straightforward prediction is that neural activity in early visual areas contributes directly to making even seemingly high-level judgments. Indeed, the model suggests that this set of features could be computed locally in these areas and projected to cortical or subcortical areas that mediate fast behavioral responses^15^. This prediction could be tested using methods similar to those in Michel *et al.*^16^, by recording neural activity in V1 in animals performing decision-making tasks with images whose curvature distribution has been synthetically modified.

## Methods Summary

The first step of our method involves defining the dictionary of templates or filters for detecting edges. We use a log-Gabor representation, which is well suited to represent a wide range of natural images^17^ (animal or non-animal). This representation gives a generic model of edges as defined by their shape, orientation, and scale. We set the parameters to match what has been reported for simple-cell responses in macaque area V1. This architecture is similar to that used by Geisler *et al.*^2^ and is detailed in the supplementary material.

The resulting dictionary of edge filters is over-complete. The linear representation would thus give an inefficient representation of the distribution of edges (and thus of the statistics of edge co-occurrences). Therefore, starting from this linear representation, we searched for the most sparse representation. Because this search is combinatorial and thus very computationally expensive, we approximated a solution using a greedy approach first proposed by Perrinet *et al.*^18^. To validate the categorization performance, we used the standard SVM library as implemented by Pedregosa *et al.*^19^. We used the Jensen–Shannon divergence distance as a metric between histograms^20^. The results of the SVM classifier are given as the F1 score to directly compare our method to that of Serre *et al.*^8^.

## Additional Information

**How to cite this article**: Perrinet, L. U. and Bednar, J. A. Edge co-occurrences can account for rapid categorization of natural versus animal images. *Sci. Rep.* **5**, 11400; doi: 10.1038/srep11400 (2015).

## Supplementary Material

Supplementary Information
