# Supplementary material for: Edge co-occurrences can account for rapid categorization of natural versus animal images
Source: Sci Rep. 2015 Jun 22;5:11400. doi: 10.1038/srep11400 (PMC4476147; doi:10.1038/srep11400)
Supplement: Supplementary Information [file srep11400-s1.pdf]

# Supplementary Information to
# *Edge co-occurrences can account for rapid categorization of natural versus animal images*


Laurent U. Perrinet[1] and James A. Bednar[2]

[1]Institut de Neurosciences de la Timone, CNRS / Aix-Marseille Université
[2]Institute for Adaptive and Neural Computation, University of Edinburgh


This article provides supplementary information related to the article, first providing supplementary detailed methods, and then presenting supplementary results mentioned in the main text.

## 1 Supplementary Methods

A method for measuring the statistics of edge co-occurrences in natural images was demonstrated by Geisler, Perry, Super & Gallogly [17]. Here we extend their method in two important ways. First, we use an over-complete, multi-scale representation of edges, which is more similar to the output of the primary visual cortex. Second, we use a synthesis model for the edge representation, so that the edges we detect are guaranteed to be sufficient to regenerate the image with a low error. Here we describe each of these procedures (see SI Figure 1), along with the construction of the statistics of edge co-occurrences (see Section 1.3) and the implementation of the classifier (see Section 1.4).

### 1.1 Linear representation of edges

The first step of our method involves defining the dictionary of templates (or filters) for detecting edges. We use a log-Gabor representation, which is well suited to represent a wide range of natural images[18]. This representation gives a generic model of edges parameterized by their shape, orientation, and scale. We set the range of these parameters to match what has been reported for simple-cell responses in macaque primary visual cortex (V1). In particular, we set the bandwidth of the Fourier representation of the filters to $1$ and $\pi/8$ respectively in log-frequency and polar coordinates to get a family of elongated and thus orientation-selective filters (see Fischer, Sroubek, Perrinet, Redondo & Cristóbal [19] and SI Figure 1 for examples of such edges). This architecture is similar to that used by Geisler, Perry, Super & Gallogly [17]. Prior to the analysis of each image, we used the spectral whitening filter described by Olshausen & Field [20] to provide a good balance of the energy of output coefficients[18,21].

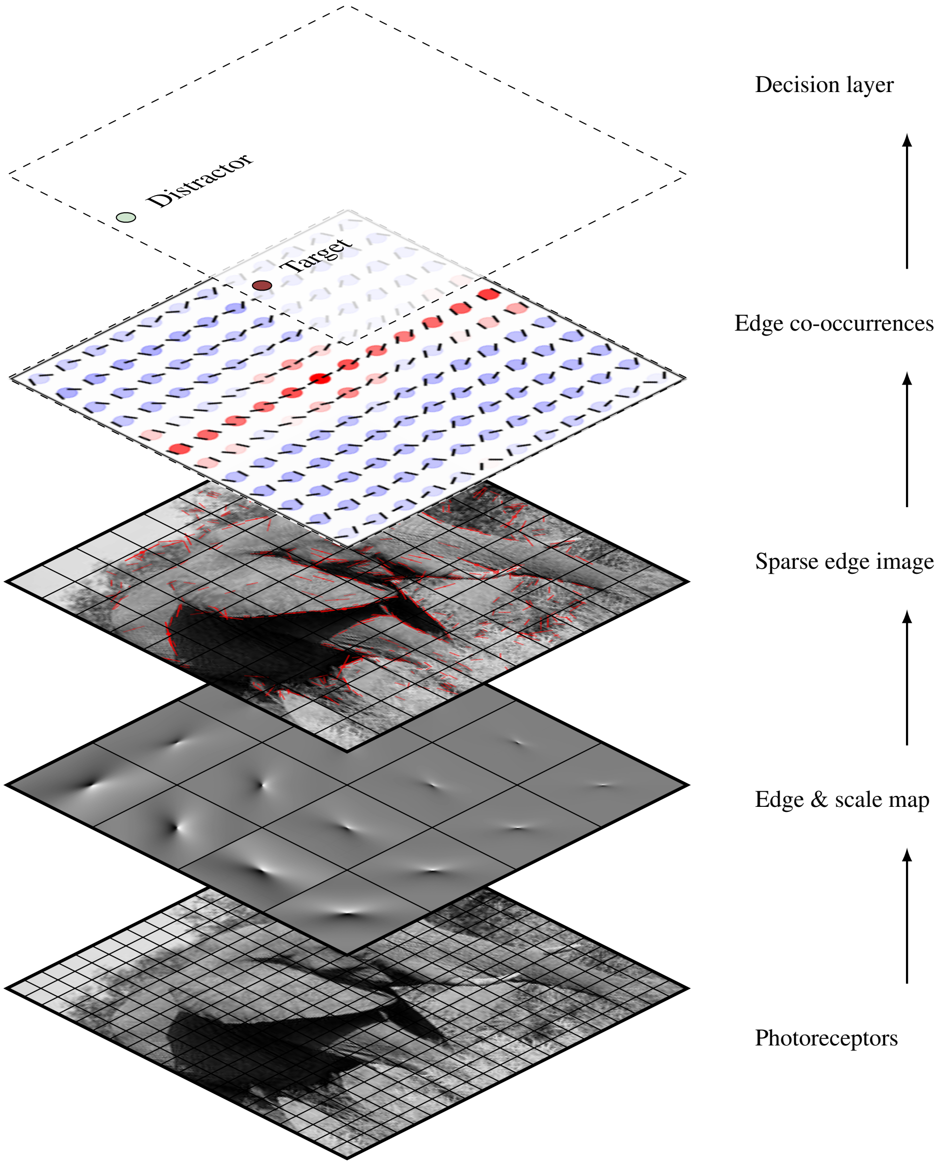


Figure 1: **Architecture of the model for edge extraction and statistics of edge co-occurrences in natural scenes.** An input image ("Photoreceptors") is first linearly convolved with a bank of filters at different orientations and scales ("Edge & scale map"), similar to the properties of cells in layer 4 of the primary visual cortex of primates. A second layer ("Sparse edge image") removes redundant information, so that only the information about the position, orientation and scale of edges remains (see example output in main text Figure 1-A). A third layer ("Edge co-occurrences") pools the different possible co-occurrences of edge configurations into a map that serves as the input of a classifier ("Decision layer").

A linear convolution model automatically provides a translation-invariant representation. Such invariance can be extended to rotations and scalings by choosing to multiplex these sets of filters at different orientations and spatial scales. Although orthogonal representations are popular for computer vision due to their computational tractability, it is desirable in our context that we have a high over-completeness in the representation to have a detailed measure of the association field. Ideally, the parameters of edges would vary in a continuous fashion, to provide relative translation, rotation, and scale invariance. We chose to have $8$ dyadic levels (that is, doubling the scale at each level) for the set of $256 \times 256$ images, with $24$ different orientations. Orientations are measured as an undirected angle in radians, in the range from $0$ to $\pi$ (but not including $\pi$). Tests with a range of different numbers of orientations and scales yielded similar results. Finally, each image is transformed into a pyramid of coefficients. This pyramid consists of approximately $4/3 \times 256^2 \approx 8.7 \times 10^4$ pixels multiplexed on $8$ scales and $24$ orientations, that is, approximately $16.7 \times 10^6$ coefficients, an over-completeness factor of about $256$.

This transform is linear and can be performed by a simple convolution repeated for every edge type. Following Fischer, Sroubek, Perrinet, Redondo & Cristóbal [19], convolutions were performed in the Fourier (frequency) domain for computational efficiency. The Fourier transform allows for a convenient definition of the edge filter characteristics, and convolution in the spatial domain is equivalent to a simple multiplication in the frequency domain. By multiplying the envelope of the filter and the Fourier transform of the image, one may obtain a filtered spectral image that may be converted to a filtered spatial image using the inverse Fourier transform. We exploited the fact that by omitting the symmetrical lobe of the envelope of the filter in the frequency domain, the output of this procedure gives a complex number whose real part corresponds to the response to the symmetrical part of the edge, while the imaginary part corresponds to the asymmetrical part of the edge (see Fischer, Sroubek, Perrinet, Redondo & Cristóbal [19] for more details). More generally, the modulus of this complex number gives the energy response to the edge (comparable to the response of complex cells in area V1), while its argument gives the exact phase. This property further expands the richness of the representation.

## 1.2 Sparse coding and validation of the edge extraction method

Because this dictionary of edge filters is over-complete, the linear representation would give a inefficient representation of the distribution of edges (and thus of edge co-occurrences) due to *a priori* correlations between coefficients. Therefore, starting from this linear representation, we searched for the most sparse representation. Minimizing the $\ell_0$ pseudo-norm (the number of non-zero coefficients) leads to an expensive combinatorial search with regard to the dimension of the dictionary (it is NP-hard). As proposed first by Perrinet, Samuelides & Thorpe [22], we may approximate a solution to this problem using a greedy approach.

In general, a greedy approach is applied when finding the best combination is difficult to solve globally, but can be solved progressively, one element at a time. Applied to our problem, the greedy approach corresponds to first choosing the single filter $\Phi_i$ that best fits the image along with a suitable coefficient $a_i$, such that the single source $a_i \Phi_i$ is a good match to the image. Examining every filter $\Phi_j$, we find the filter $\Phi_i$ with

the maximal correlation coefficient, where:

$$i = \text{argmax}_j \left( \left\langle \frac{\mathbf{I}}{||\mathbf{I}||}, \frac{\Phi_j}{||\Phi_j||} \right\rangle \right), \tag{1}$$

$\langle\cdot,\cdot\rangle$ represents the inner product, and $||\cdot||$ represents the $\ell_2$ (Euclidean) norm. Since filters at a given scale and orientation are generated by a translation, this operation can be efficiently computed using a convolution, but we keep this notation for its generality. The associated coefficient is the scalar projection:

$$a_i = \left\langle \mathbf{I}, \frac{\Phi_i}{||\Phi_i||^2} \right\rangle \tag{2}$$

Second, knowing this choice, the image can be decomposed as

$$\mathbf{I} = a_i \Phi_i + \mathbf{R} \tag{3}$$

where $\mathbf{R}$ is the residual image. We then repeat this 2-step process on the residual (that is, with $\mathbf{I} \leftarrow \mathbf{R}$) until some stopping criterion is met. Note also that the norm of the filters has no influence in this algorithm on the choice function or on the reconstruction error. For simplicity and without loss of generality, we will thereafter set the norm of the filters to $1$: $\forall j, ||\Phi_j|| = 1$. Globally, this procedure gives us a sequential algorithm for reconstructing the signal using the list of sources (filters with coefficients), which greedily optimizes the $\ell_0$ pseudo-norm (i.e., achieves a relatively sparse representation given the stopping criterion). The procedure is known as the Matching Pursuit (MP) algorithm[23], which has been shown to generate good approximations for natural images[24].

For this work we made two minor improvements to this method: First, we took advantage of the response of the filters as complex numbers. As stated above, the modulus gives a response independent of the phase of the filter, and this value was used to estimate the best match of the residual image with the possible dictionary of filters (Matching step). Then, the phase was extracted as the argument of the corresponding coefficient and used to feed back onto the image in the Pursuit step. This modification allows for a phase-independent detection of edges, and therefore for a richer set of configurations, while preserving the precision of the representation.

Second, we used a "smooth" Pursuit step. In the original form of the Matching Pursuit algorithm, the projection of the Matching coefficient is fully removed from the image, which allows for the optimal decrease of the energy of the residual and allows for the quickest convergence of the algorithm with respect to the $\ell_0$ pseudo-norm (i.e., it rapidly achieves a sparse reconstruction with low error). However, this efficiency comes at a cost, because the algorithm may result in non-optimal representations due to choosing edges sequentially and not globally. This is often a problem when edges are aligned (e.g. on a smooth contour), as the different parts will be removed independently, potentially leading to a residual with gaps in the line. Our goal here is not to get the fastest decrease of energy, but rather to provide a good representation of edges along contours. We therefore used a more conservative approach, removing only a fraction (denoted by $\alpha$) of the energy at each pursuit step (for MP, $\alpha = 1$). We found that $\alpha = 0.5$ was a good compromise between rapidity and smoothness. One consequence of using $\alpha < 1$ is that, when removing energy along contours, edges can overlap; even so, the correlation is invariably reduced. Higher and smaller values of $\alpha$ were also tested, and gave classification results similar to those presented here.

In summary, the whole learning algorithm is given by the following nested loops in pseudo-code:

1. draw a signal $\mathbf{I}$ from the database; its energy is $E = ||\mathbf{I}||^2$,
2. initialize sparse vector $\mathbf{s}$ to zero and linear coefficients $\forall j, a_j =< \mathbf{I}, \Phi_j >$,
3. while the residual energy $E = ||\mathbf{I}||^2$ is above a given threshold do:
   (a) select the best match: $i = \text{ArgMax}_j |a_j|$, where $|\cdot|$ denotes the modulus,
   (b) increment the sparse coefficient: $s_i = s_i + \alpha \cdot a_i$,
   (c) update residual image: $\mathbf{I} \leftarrow \mathbf{I} - \alpha \cdot a_i \cdot \Phi_i$,
   (d) update residual coefficients: $\forall j, a_j \leftarrow a_j - \alpha \cdot a_i < \Phi_i, \Phi_j >$,
4. the final non-zero values of the sparse representation vector $\mathbf{s}$, give the list of edges representing the image as the list of couples $(i, s_i)$, where $i$ represents an edge occurrence as represented by its position, orientation and scale.

This class of algorithms gives a generic and efficient representation of edges, as illustrated by the example in main text Figure 1-A. We also verified that the dictionary used here is better adapted to the extraction of edges than Gabors[18]. The performance of the algorithm can be measured quantitatively by reconstructing the image from the list of extracted edges. Measuring the ratio of extracted energy in the images, $N = 1024$ edges were enough to extract an average of $95\%$ of the energy of $256 \times 256$ images on all sets of images. All simulations were performed using Python (version 2.6) with packages NumPy (version 1.6.2) and SciPy (version 0.7.2)[25] on a cluster of Linux computing nodes. Visualization was performed using Matplotlib (version 1.1.0)[26]. All scripts are available upon request to the corresponding author.

## 1.3 Histogram of edge co-occurrences and geometrical symmetries

As in Geisler, Perry, Super & Gallogly [17], we will now measure the statistics of edge co-occurences using the definitions presented in main text Figure 1-B. We will be using the edges that we extracted following the method presented in the previous section. Note that since we are considering only relative orientations, co-occurrences have several geometrical symmetries: if an occurrence exists for a configuration $(\phi, \theta)$, then it exists also for $(\phi + \pi, \theta + \pi)$ (considering other orientations of the first edge by a rotation of $\pi$ radian), $(\phi + \pi - \theta, \pi - \theta)$ (swapping both edges) and $(\phi - \theta, -\theta)$ (rotation of $\pi$ radians). For that reason, it is convenient to define $\psi = \phi - \theta/2$ (see main text Figure 1-B). As $\psi$ is symmetric with respect to the choice of the reference edge, for a configuration $(\psi, \theta)$, we have also the following symmetries $(\psi + \pi, \theta + \pi)$, $(\psi + \pi, \pi - \theta)$ and $(\psi, -\theta)$.

Geometrically, $\psi$ is the angle between (1) the mediator of the segment joining the edges' centers and (2) the line joining the center of this segment to the intersection of the normal of the segments (see main text Figure 1-B). Note that for a pair of edges on a common circle, we have $\phi = \theta/2$, that is, $\psi = 0$ (see the central vertical axis in main text Figure 2). This convention gives a simpler representation of circularities (for similar approaches see refs.[27–29]), and $\psi$ will denote the difference of azimuth in the rest of the paper. Colinearity ($\theta = \psi = 0$) and other parallel edges ($\theta = 0$) are represented on the central horizontal axis of main text Figure 2.

Main text Figure 2 shows a horizontal periodicity of $\pi$, a vertical periodicity of $\pi$, and a mirror symmetry around the horizontal axis $\theta = 0$. Furthermore, we observed that there is typically an axial symmetry with respect to the mediator (that is, in any given image set, a configuration $(\psi, \theta)$ is as likely as $(-\psi, \theta)$), corresponding to mirror

versions of images around the vertical axis $\psi = 0$. Due to the finite number of measurements, empirical results (see SI Figure 2-A, or figure 3-C from Geisler, Perry, Super & Gallogly [17]) will of course not have perfect symmetry in practice. Since $\phi$, $\psi$ and $\theta$ are angles defined for instance between $-\pi$ and $\pi$, these symmetries allows us to consider only a single quadrant (by convention the upper right, that is $-\pi/2 < \phi \leq \pi/2$, $-\pi/2 < \psi \leq \pi/2$ and $0 \leq \theta \leq \pi/2$), the rest being inferred by the above relations. We used this additional looser type of symmetry only for simplifying visualizations, not for the underlying calculations.

### 1.4 Classification method

To validate the categorization performance, we used the standard SVM library as implemented by Pedregosa *et al.* [30]. First, we randomly divided each database into a training and a testing sub-set. In order to evaluate a distance between histograms, we used the Jensen–Shannon divergence distance as a metric between histograms[31]. Thus, we directly supplied a precomputed Gram matrix of the distance between each pair of histograms to the classifier. We used the default parameters of the method. Other choices of parameters or of kernels (that is, between linear, radial basis functions, or precomputed) gave qualitatively similar results. Fitting the classifier to the training set was done using an automatic line search algorithm from the same library [30]. The results of the SVM classifier are usually given as the precision, recall, or F1 score. Here we used the latter to directly compare our method to that of Serre, Oliva & Poggio [32]. This process was cross-validated 20 times by drawing new training and testing sets. Using these different trials, we could measure the variability of the F1 score. The variability was always in the range of $\approx 4\%$.

## 2 Supplementary Results

Our goal is to study how the statistics of edge co-occurrence vary across three image categories, so we defined three testing databases. The first two consist of the image databases ($600$ images each)[1] used by Serre, Oliva & Poggio [32], which contain either animals at different close-up views in a natural setting (which we call "animal image"), or natural images without animals, which we call "non-animal natural images". A third database for comparison consists of self-acquired images from a biology laboratory setting, containing $600$ indoor views of furniture, windows, and doors and cages in which animals are reared (which we call "man-made images").

### 2.1 First-order statistics

One obvious candidate representation for categorization is the first-order statistics of edges. In natural images, edges are more frequently aligned to the cardinal axes, especially for man-made scenes, as has been reported and modeled previously by others[33]. As the spectrum of edges is localized in the Fourier domain[19], the representation of first-order statistics of edges is equivalent to using the amplitude spectrum obtained by Fourier analysis of the raw image. The spectral signature of scenes has previously been used by computational models to infer scene categories[34,35]), and the human visual system could take advantage of these low-level natural image statistics. To compare with these previous results, we computed first-order statistics on the sparse representation

[1]Publicly available at *http://cbcl.mit.edu/software-datasets/serre/SerreOlivaPoggioPNAS07*.

described in the methods section. The histograms yielded similar results to those found on the amplitude spectrum of the raw image[34,35]. However, these first-order statistics, while tending to be different on average for different scene categories, are also highly variable within each category. The first-order histogram is highly dependent on geometrical constraints that are independent of the scene category, like the field of view (close-up or full-field view) or the orientation relative to the horizon, and we show below that they are not particularly reliable for classifying individual images into these different categories (see Table in main text). Most importantly, these results fall to chance level with a rotation of the image or to changes in the spectral envelope, in contradiction with behavioral results[36,37]. First-order statistics are therefore a relatively poor indicator of scene category.

## 2.2 Statistics of edge co-occurrences

Statistics of edge co-occurrences could represent a better alternative. Indeed, image semantics seem to depend not on spatial-frequency amplitude, but rather on phase information[38], which is also essential for discriminating textures[27]. Like Geisler, Perry, Super & Gallogly [17], we have chosen to compute the histogram of edge co-occurrences, that is, the frequentist probability of an edge knowing a reference edge (yielding $N \cdot (N-1)/2 = 523776$ samples per image when using $N = 1024$ edges as we do here). This histogram is a 4-dimensional function of (1) the distance $d$ between two edges, (2) the difference of azimuth $\phi$ of the center of one edge with respect to the position and orientation of the reference edge, (3) the difference of orientation $\theta$ between the two edges, and (4) the ratio of edge scales $\sigma$ (see diagram in main text Figure 1-B). By definition of our representation, this set of statistics is independent of translations, rotations in the image plane, and scalings.

First, we replicated the results of Geisler, Perry, Super & Gallogly [17] on a set of natural images to validate our procedure, from the edge representation to the extraction. We computed similar projections of the histograms as in Geisler, Perry, Super & Gallogly [17] and found qualitatively similar results despite the different datasets and methods used. As in Geisler, Perry, Super & Gallogly [17], the finding is that in natural images, edges are more likely to be organized in co-linear or parallel textures (see SI Figure 2-A) and along co-circular paths with a prior for low curvatures (see SI Figure 2-B). What is more interesting is that when using images from different environments such as a man-made environment (brownish edges in the figure), one finds a different pattern, where co-linearity dominates. This qualitative difference clearly indicates that the statistics of edge co-occurrences differ between databases. However, the precise way in which these sets differ is not necessarily clear, which will be analyzed in the next section.

## 2.3 Separating relevant variables in edge co-occurrences' statistics

The full set of second-order statistics is a function of four variables, which is difficult to plot and analyze, and so we considered whether it was possible to factorize this function into components that can be analyzed separately. We computed the mutual information of the joint probability with the 12 possible combinations of the factorizations of $p(d, \phi, \theta, \sigma)$. This calculation gives different Kullback-Leibler distances[31] in bits between the factorizations and the original function, in order to measure the independence of each hypothesized factorization. For all sets of images, four good candidate factorizations emerge (see Table 1): $p(\theta, \sigma, d) \cdot p(\phi)$, $p(\sigma, d, \phi) \cdot p(\theta)$, $p(\phi) \cdot p(\theta) \cdot p(\sigma, d)$ and

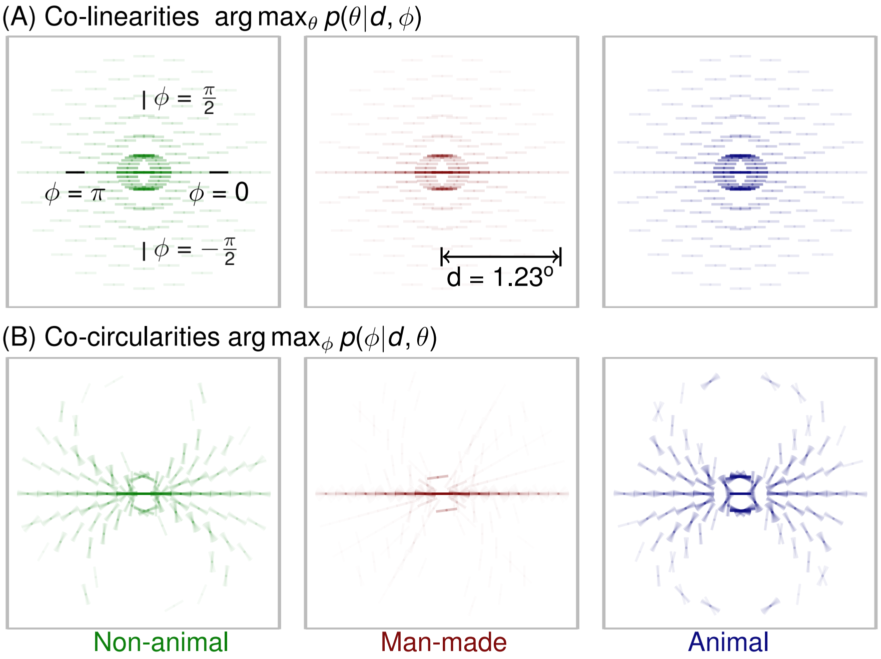


Figure 2: **Edge co-occurrences in different categories of natural scenes.** From the edge representation (see main text Figure 1-A), it is possible to compute the statistics of edge co-occurrences (see main text Figure 1-B) for each set of images. Here we show a replication of the results from[17] for non-animal images (in greenish color), extended to man-made images (in brownish color) and animals (in blueish color). The statistics of edge co-occurrence correspond to a 4-dimensional histogram reporting, relative to a given edge, distance $d$, difference of azimuth $\phi$, difference of orientation $\theta$, and ratio of scale $\sigma$ as a single function $p(d, \phi, \theta, \sigma)$. (A) As in[17], we can project this function to see the most probable orientation difference knowing any possible position (determined by the distance $d$ and difference of azimuth $\phi$) relative to the reference edge (i.e., $\arg\max_\theta p(\theta|d, \phi)$). Note that we marginalize here relative to the scale $\sigma$, but that we observed that each individual scale behaved similarly, as expected by the invariance to zooms of these statistics. The results show that at every position, the most probable orientation of an edge is always parallel to the reference edge, reflecting a primary trend for parallel textures and patterns to occur in images of all categories. (B) Additionally, as in ref.[17], we can project this function onto other axes to show the most likely azimuth for each orientation difference and each given distance (i.e., $\arg\max_\phi p(\phi|d, \theta)$). The results show that when the difference of orientation $\theta$ is nonzero, it tends to be for co-circular contours (so-called "good completions") in natural images[28] and in natural images containing an animal, while straight lines dominate in man-made images. In both plots, the value of the maximum probability relative to the central reference edge is represented by the transparency of the edge shown and relative to the reference edge in the center of the plot. These results replicate ref.[17] and suggest that statistics of edge co-occurrences in image categories contain important information that could be used as a prior. In addition, while parallel textures dominate for all categories in (A), the pattern in (B) clearly differs qualitatively between databases, though further analysis in subsequent figures will be required to demonstrate these differences quantitatively.

$p(\phi, \theta) \cdot p(\sigma, d)$). An emergent pattern is that we may separate the characteristic angles ($\phi$ and $\theta$, individually or together) from distance-related statistics ($d$ and $\sigma$). The distribution $p(d, \sigma)$ proved to be quite similar across the different classes of images, as it is more characteristic of the overall configuration of the scene than of the objects within it (see SI Figure 3).

Table 1: **Testing the relative dependence of all histogram variables.** Given the histogram of second order statistics $p(d, \phi, \theta, \sigma)$ (which we will subsequently denote simply $p$), we have computed its entropy and the Kullback-Leibler (KL) divergence with all combinations of the factorization of the variables $d$, $\phi$, $\theta$ and $\sigma$ (in bits, averaged over all images from all categories). Entropy is the KL divergence of $p$ with the uniform distribution and serves as an upper bound. The KL measure is positive and only the full statistics can achieve $KL = 0$ (by Gibbs' inequality), but the next four factorizations cause very little error, and are thus good candidates. For these factorizations, we may separate the characteristic angles ($\phi$ and $\theta$, individually or together) from distance-related statistics ($d$ and $\sigma$) that are characteristic of the configuration of the view. The remaining factorizations are much more different from the full distribution, and thus will not be considered further.

| $Entropy(p)$ | $\approx 0.724$ |
|---|---|
| $\mathrm{KL}(p\|\|p)$ | $= 0$ |
| $\mathrm{KL}(p\|\|p(\theta, \sigma, d) \cdot p(\phi))$ | $\approx 0.001$ |
| $\mathrm{KL}(p\|\|p(\sigma, d, \phi) \cdot p(\theta))$ | $\approx 0.004$ |
| $\mathrm{KL}(p\|\|p(\phi, \theta) \cdot p(\sigma, d))$ | $\approx 0.004$ |
| $\mathrm{KL}(p\|\|p(\phi) \cdot p(\theta) \cdot p(\sigma, d))$ | $\approx 0.006$ |
| $\mathrm{KL}(p\|\|p(\phi, \theta, \sigma) \cdot p(d))$ | $\approx 0.057$ |
| $\mathrm{KL}(p\|\|p(\theta, \sigma) \cdot p(d, \phi))$ | $\approx 0.058$ |
| $\mathrm{KL}(p\|\|p(\theta, \sigma) \cdot p(d) \cdot p(\phi))$ | $\approx 0.058$ |
| $\mathrm{KL}(p\|\|p(d, \phi, \theta) \cdot p(\sigma))$ | $\approx 0.059$ |
| $\mathrm{KL}(p\|\|p(\sigma, \phi) \cdot p(d, \theta))$ | $\approx 0.060$ |
| $\mathrm{KL}(p\|\|p(\phi, \theta) \cdot p(\sigma) \cdot p(d))$ | $\approx 0.060$ |
| $\mathrm{KL}(p\|\|p(d, \phi) \cdot p(\theta) \cdot p(\sigma))$ | $\approx 0.061$ |
| $\mathrm{KL}(p\|\|p(d) \cdot p(\phi) \cdot p(\theta) \cdot p(\sigma))$ | $\approx 0.061$ |

Let us now focus on the map of angle configurations $p(\phi, \theta)$: This can be reduced to 2 dimensions, so that we can plot this probability as a "chevron map" $p(\psi, \theta)$. Each chevron corresponds to a possible configuration of the angles $\psi = \phi - \theta/2$ and $\theta$. Such a map is shown in main text Figure 2 with the saturation of the colored circle indicating the frequency of occurrence. The chevron map spans each possible chevron configuration, i.e., for all possible difference of azimuth values $\psi$ on the horizontal axis and difference of orientation $\theta$ on the vertical axis. Red denotes more frequent than a uniform-probability reference, while blue denotes less frequent.

Main text Figure 3 shows how the chevron map differs for the other two datasets, now relative to the map computed for the non-animal dataset. A first observation is that main text Figure 3 shows the configuration in a more compact fashion than SI Figures 2-A and 2-B. In man-made versus natural non-animal environments, there is a significant excess of parallel and co-linear edges, with a maximum for the co-linear co-

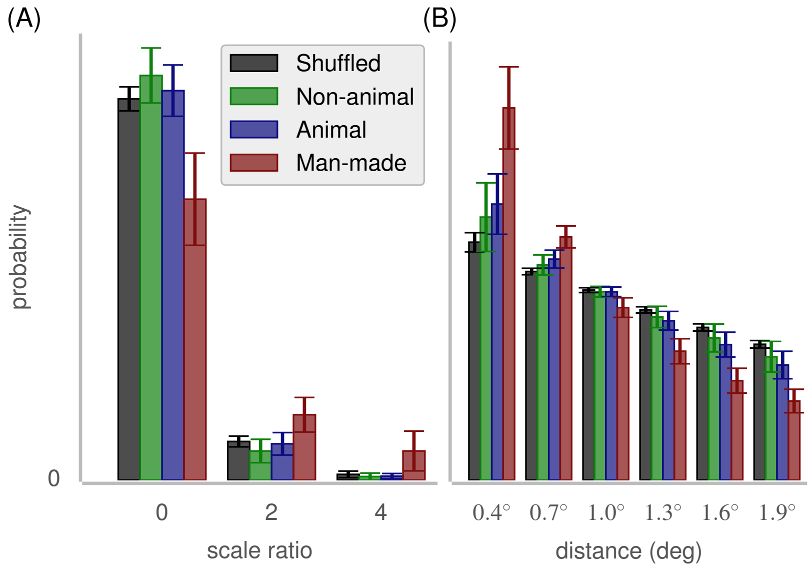


Figure 3: **Configuration variables are not significantly different across categories.** An independence analysis shows that in natural images, the statistics of edge co-occurrences can be factorized into independent components $p(\psi, \theta) \cdot p(\sigma, d)$ (see text). To show the shape of $p(\sigma, d)$, we plot in (A) the distribution of scale ratios $p(\sigma)$ and in (B) the distribution of distances to a reference edge $p(d)$. Counts are plotted for each dataset in the colors indicated (blue for non-animal, green for animals and red for man-made), along with the statistics obtained after shuffling each edge variable (in black). The bar heights allow comparison across categories, while the error bars indicate variation within each category. We created a novel set by taking the extracted edges from the set of non-animal images, then shuffling the position of their centers ("shuffled set"), such that first-order information on orientation and scale is kept while all second-order statistical information (which relies on relative positions) is lost. In (A) there is an overall decrease in probability with increasing difference in scale similar to that in the shuffled case, which is due to the finite number of scales. The distribution is consistent across all databases, with variability comparable within and between databases, and thus the scale differences are not useful for categorizing the class of an image. Similarly, (B) shows that edges are relatively clustered, with other edges significantly more likely to be closer to a given reference edge (with a maximum of about 25% more probable than in the shuffled case at the shortest range). The results for shuffled images show that there is a bias due to the finite size of images. For non-shuffled images, the change in probability with distance is mainly due to a prior preference for a clustering of edges. This distribution is consistent with scenes consisting mostly of small objects, as is well described by the dead-leaves model[39]. Again, the variation within each database in (B) is high relative to the variation between them, and so the distances are also not informative about the image class.

occurrence being about 2 times more likely than in natural non-animal images. Interestingly, in animals versus non-animal scenes, there is a relative excess of co-circular and converging configurations, with a maximum being about 1.2 times more likely

than in non-animal images. Note also a significant decrease for some configurations for man-made images than for other non-animal images (with a minimum being about 0.6 times less likely). This last point is consistent with the observation from ref.[29] that significant relationships may be either facilitating (for instance to group co-linear edges), or suppressive, to rule out some configurations as *a priori* less probable.

In order to quantitatively assess the qualitative differences that we observe in the chevron maps, we built a simple classifier to measure if this representation is sufficient to categorize different image categories. Such a finding would suggest that information contained in the statistics of edge co-occurrence in natural scenes may be used instead of or alongside a hierarchical analysis of the visual scene, when making a quick judgment as in rapid-categorization tasks.

## 2.4 Categorization of images using edge co-occurrences

As described in the main text, we consider whether alternative low-level representations could be more successful than the large set of such representations tested by Serre, Oliva & Poggio [32]. For each individual image, we constructed a vector of features as either (FO) the histogram of first-order statistics, (SO) the full histogram of edge co-occurrences, or (CM) the histogram $p(\psi, \theta)$ corresponding to the chevron map. To compare the representational power of each type of feature vector, we gathered these vectors for each different class of images and tested a standard linear Support Vector Machine (SVM) classification algorithm, as described in Section 1.4. Our results can be compared directly to those of Serre, Oliva & Poggio [32], who used the same classifier on both the last level of their hierarchical representation (successfully), and directly on the raw images (unsuccessfully). They can also be compared with the other unsuccessful low-level representations tested by Serre, Oliva & Poggio [32], such as the mean luminance, a single-template SVM classifier, texton features, global (context) features, or the output of their model V1 complex cell layer.

As a control, we also used the nearest neighbor (that is, 2-means) classifier. I.e., for any image, we computed the distance to the average histogram (centroid) for each class. Using a threshold, one can decide which centroid is closer and classify the image to its closest centroid. Averaged over all test images, this procedure gives a quantitative measure of the compromise between correct hits and false alarms with respect to the threshold; the measure is called the Receiver Operating Characteristic (ROC). The final result is computed as the Area Under the Curve (AUC). Globally, this method obtained qualitatively similar results compared to the SVM algorithm. However, the SVM algorithm performed slightly better in the vast majority of cases.

Figure 4 in the main text gives the performance of different categorizations for the three types of representations, where several patterns can be seen. First, databases that are qualitatively different (such as non-animal versus man-made images) are very well categorized, with accuracy over 98% when using the full statistics of edge co-occurrences. For images of man-made objects this result may be obvious, given their prevalence of highly regular co-linear edges. It is perhaps more surprising, particularly given the claims from Serre, Oliva & Poggio [32] that low-level cues were unlikely to work, that we also achieved quite high, robust performance for classifying images containing animals versus other natural (non-animal) images. Second, it is interesting that results for the chevron map are almost as high as when using the full probabilities, confirming that the performance of the classifier comes primarily from a geometrical feature rather than a viewpoint-dependent feature (such as the scale of edges). This confirms our claim that configuration and geometrical variables are relatively indepen-

dent (see SI Figure 3). These results were also applied to a novel set of non-animal, animal and man-made images, giving similar results (see SI Figure 5).

Our hypothesis is that classifying images containing animals versus other natural non-animal images was successful because of the higher prevalence of co-circular and converging contours in images containing animals (see main text Figure 3). If humans are using similar mechanisms, their performance should decrease with the size of the animal relative to its background, because the statistics of the background will become more prominent. Figure 4 shows that the model performance closely tracks that of human observers on the image sub-categories defined by Serre, Oliva & Poggio [32] based on the closeness of the animals. Performance for the first-order edge statistics does not similarly vary, suggesting that they reflect incidental differences in the image databases that are not due to the actual presence of the animal. Performance for all the models is much higher on far images (where the animal is very small) than for humans, presumably because the task did not allow the humans to move their eyes during the presentation, and the animals were not necessarily at the center of fixation.

## 2.5 Robustness to noise, translation and rotations

Note that by definition, our measure of the statistics of edge co-occurrence is invariant to translations, scalings, and rotations in the plane of the image (unlike the first-order statistics). Thus, despite any of these transformations, one can efficiently differentiate between images from different categories. This property makes it possible to explain the rather unintuitive result that ultra-rapid categorization in humans is relatively independent to rotations[36] (see also the supplementary information of Serre, Oliva & Poggio [32]). We also performed the same classification where images from both databases were perturbed by adding independent Gaussian noise to each pixel such that signal-to-noise ratio was halved. As can be seen in Figure 4 of the main text and SI Figure 5, results are degraded but qualitatively similar. Edge extraction in the presence of noise may result in false edges, but the underlying statistics of the chevron maps are still robustly captured, thanks to the high number of co-occurrences that are measured.

# References


17. Geisler, W. S., Perry, J. S., Super, B. J. & Gallogly, D. P.: Edge co-occurence in natural images predicts contour grouping performance. *Vision Research* **41**, 711–24 (2001).

18. Fischer, S., Redondo, R., Perrinet, L. U. & Cristóbal, G.: Sparse approximation of images inspired from the functional architecture of the primary visual areas. *EURASIP Journal on Advances in Signal Processing* **2007**, 090727–122 (2007).

19. Fischer, S., Sroubek, F., Perrinet, L. U., Redondo, R. & Cristóbal, G.: Self-invertible 2D log-Gabor wavelets. *International Journal of Computer Vision* **75**, 231–246 (2007).

20. Olshausen, B. A. & Field, D. J.: Sparse coding with an overcomplete basis set: A strategy employed by V1? *Vision Research* **37**, 3311–3325 (1997).

21. Perrinet, L., Samuelides, M. & Thorpe, S.: Coding Static Natural Images Using Spiking Event Times: Do Neurons Cooperate? *IEEE Transactions on Neural Networks* **15**, 1164–1175 (2004).

22. Perrinet, L., Samuelides, M. & Thorpe, S.: Sparse spike coding in an asynchronous feed-forward multi-layer neural network using matching pursuit. *Neurocomputing* **57**, 125–134 (2004).

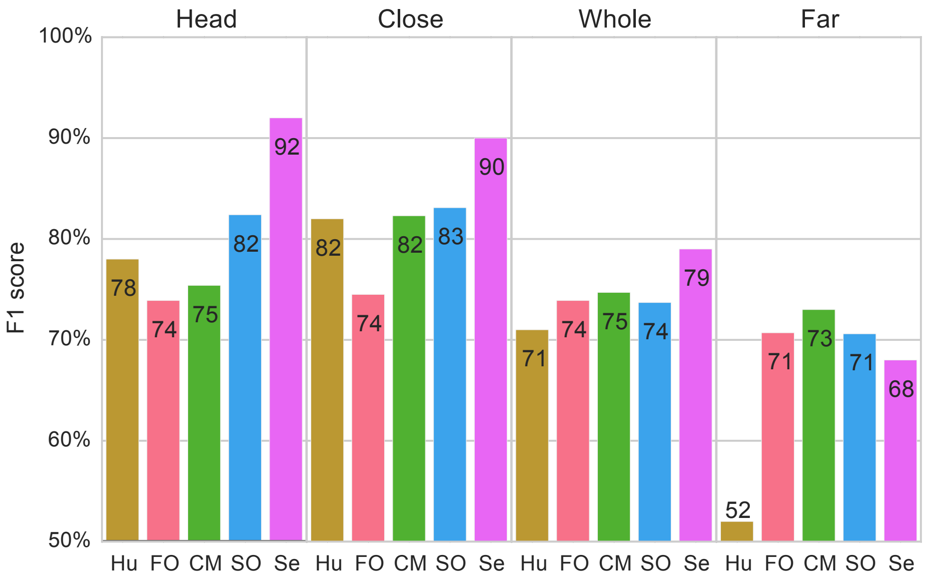


Figure 4: Classification results (F1 score) within image subcategories, given for (Hu) human observers, (Se) the hierarchical model of Serre, Oliva & Poggio [32], and (FO, CM, and SO) the representations generated by our models. Several patterns can be observed in these results. (1) Human performance falls to near chance level for detecting the 'Far' animals, which is presumably because the relatively small animals in these images are not necessarily at the point of gaze, and in these experiments there is no time for making eye movements. Conversely, all of the models outperform humans under these conditions, because they are provided the entire image. (2) The FO model performance does not vary significantly with the size of the animal, suggesting that it is based on incidental features of the image datasets rather than the actual presence of an animal. (3) The CM and SO models track the patterns of performance for humans quite well, apart from the high performance for far images that all the models have. (4) The Serre et al. model significantly outperforms humans in each category, which would be expected if it is modelling hierarchical processing strategies not available to humans in the rapid time scales of these experiments.

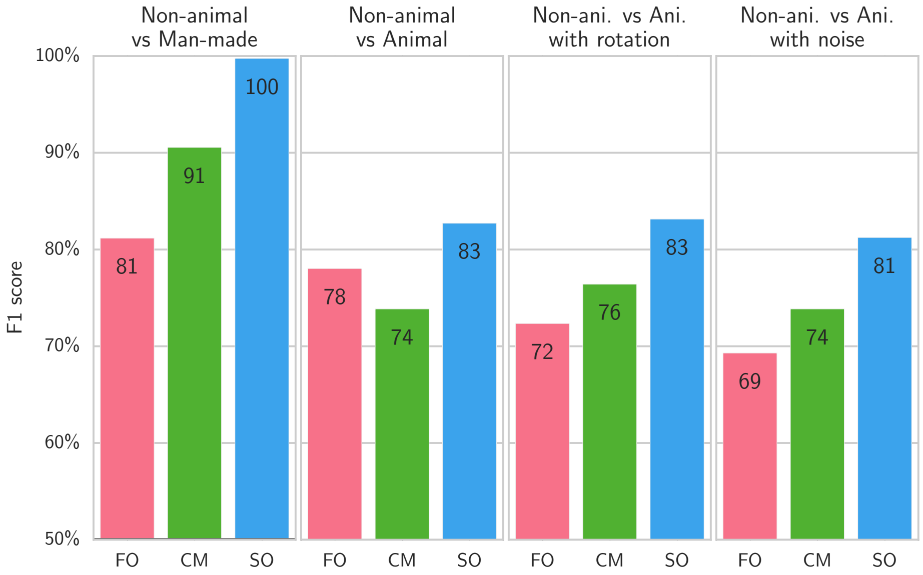


Figure 5: Classification results on other datasets. To test the generality of the results presented in the main text, we applied the same method to additional collections of images from other sources. These datasets were of similar size (600 images per database, each of size $244 \times 244$ pixels) as in the main text, but did not contain any of the same images as in the previous results. The non-animal and animal datasets originated from the same source as [40, 41], while the man-made dataset was a series of images taken in a different laboratory environment, from a previous study [42]. As for figure 4 of the main text, for each individual image, we constructed a vector of features as either (FO) the histogram of first-order edge statistics, (CM) the two-dimensional chevron map subset of the second-order statistics (see Figure 3), or (SO) the full, four-dimensional second-order statistics. We gathered these vectors for each different class of images and report here the results of the SVM classifier using an F1 score (where 50% represents chance level). Results are similar to those presented in the main text, except that for these datasets the animals are well categorized (F1 score= 78%) using first-order statistics alone. This appears to be due to a bias in that database's selection of non-animal images, which include wide landscapes and man-made scenes with easily detectable cardinal orientations. However, the first-order classification results decrease when a random rotation has been applied to the image (F1 score= 72%), while second-order features are insensitive to such perturbation (as for humans[36]) and nearly insensitive to added noise, and thus they are a reliable indicator of image category.

23. Mallat, S. & Zhang, Z.: Matching Pursuit with time-frequency dictionaries. *IEEE Transactions on Signal Processing* **41**, 3397–3414 (1993).

24. Perrinet, L. U.: Role of Homeostasis in Learning Sparse Representations. *Neural Computation* **22**, 1812–1836 (2010).

25. Oliphant, T. E.: Python for Scientific Computing. *Computing in Science and Engineering* **9**, 10–20 (2007).

26. Hunter, J. D.: Matplotlib: A 2D Graphics Environment. *Computing in Science and Engineering* **9**, 90–95 (2007).

27. Motoyoshi, I. & Kingdom, F. A. A.: The role of co-circularity of local elements in texture perception. *Journal of Vision* **10**, 3+ (2010).

28. Sigman, M., Cecchi, G. A., Gilbert, C. D. & Magnasco, M. O.: On a common circle: Natural scenes and Gestalt rules. *Proceedings of the National Academy of Sciences* **98**, 1935–1940 (2001).

29. Hunt, J. J., Bosking, W. H. & Goodhill, G. J.: Statistical structure of lateral connections in the primary visual cortex. *Neural Systems & Circuits* **1**, 3+ (2011).

30. Pedregosa, F. *et al.:* Scikit-learn: Machine Learning in Python. *Journal of Machine Learning Research* **12**, 2825–2830 (2011).

31. Cha, S.-H. & Srihari, S. N.: On measuring the distance between histograms. *Pattern Recognition* **35**, 1355–1370 (2002).

32. Serre, T., Oliva, A. & Poggio, T.: A feedforward architecture accounts for rapid categorization. *Proceedings of the National Academy of Sciences* **104**, 6424–6429 (2007).

33. Girshick, A. R., Landy, M. S. & Simoncelli, E. P.: Cardinal rules: visual orientation perception reflects knowledge of environmental statistics. *Nature Neuroscience* **14**, 926–932 (2011).

34. Oliva, A. & Torralba, A.: Modeling the Shape of the Scene: A Holistic Representation of the Spatial Envelope. *International Journal of Computer Vision* **42**, 145–175 (2001).

35. Torralba, A. & Oliva, A.: Statistics of natural image categories. *Network* **14**, 391–412 (2003).

36. Crouzet, S. M. & Serre, T.: What are the visual features underlying rapid object recognition? *Frontiers in Psychology* **2**, 326+ (2011).

37. Gaspar, C. M. & Rousselet, G. A.: How do amplitude spectra influence rapid animal detection? *Vision Research* **49**, 3001–3012 (2009).

38. Oppenheim, A. & Lim, J.: The importance of phase in signals. *Proceedings of the IEEE* **69**, 529–541 (1981).

39. Pitkow, X.: Exact feature probabilities in images with occlusion. *Journal of Vision* **10** (2010).

40. Kirchner, H. & Thorpe, S. J.: Ultra-rapid object detection with saccadic eye movements: Visual processing speed revisited. *Vision Research* **46**, 1762–1776 (2006).

41. Crouzet, S. M., Kirchner, H. & Thorpe, S. J.: Fast saccades toward faces: Face detection in just 100 ms. *Journal of Vision* **10** (2010).

42. Rudiger, P. P., Stevens, J.-L., Talluri, B. C., Perrinet, L. & Bednar, J.: Relationship between natural image statistics and lateral connectivity in the primary visual cortex. *Proceedings of COSYNE* (2014).
